# Osiris^++^: hierarchical representations for robotic-enabled precision agriculture

**DOI:** 10.3389/frobt.2026.1732004

**Published:** 2026-02-26

**Authors:** Adam Mukuddem, Adam Speed-Andrews, Thabisa Maweni, Imannuel Nanyaro, Ritvik Sojen, Venny Hsiao, Paul Amayo

**Affiliations:** African Robotics Unit, University of Cape Town, Cape Town, South Africa

**Keywords:** field robotics, human–robot interaction, perception, precision agriculture, robotics

## Abstract

There has been significant development in agricultural robotics over the past few years in the pursuit of optimising efficiency and addressing issues such as labour shortages and humans performing hazardous and arduous tasks. Despite this, human–robot interaction in the agricultural sector remains largely unchanged, often requiring technical expertise, which hinders wide-scale adoption. This problem is particularly pronounced in the African context, where limited technical exposure and linguistic diversity pose significant barriers to the adoption of these technologies. While alternative means for human–robot collaboration have been developed, these methods are currently limited to indoor structured environments. In this work, we introduce Osiris++, a flexible approach designed to allow seamless communication between robots and humans on an array of precision agriculture tasks. We validate and evaluate the performance of Osiris++ in real-world agricultural environments, demonstrating that the system can create accurate and useful scene graphs that aid in solving the assigned tasks. This paves the way for the possibility of allowing natural language instructions, including those in African languages, to be issued to robots within the agricultural sector.

## 1 Introduction

There has been a continuous global interest in human–robotic collaboration within the robotics community, with this approach maximising the unique capabilities of both humans and robots and minimising the perceived threat of human displacement in the workplace (Ajoudani et al., 2018). Effective robot–human interfaces remain an open problem, particularly in the agricultural sector, where many workers have minimal technological training (Lytridis et al., 2021) and, therefore, require user-friendly interfaces.

Conventional farming methodologies are characterised by high labour intensity and, in the face of increasing shortages of skilled labour and increasing costs, may prove inadequate for achieving optimal efficiency and productivity in agricultural practices (Bai et al., 2023). Agricultural vehicle operators must function with considerable precision to reduce areas of omission and overlap. This task imposes significant demands on humans, who must constantly supervise the traversed paths and make real-time trajectory adjustments (Bai et al., 2023). Although autonomous agricultural robots and vehicles have the potential to perform such demanding tasks and mitigate the efficacy and productivity concerns associated with human labour (Duckett et al., 2018), the development of a comprehensive autonomous navigation system remains an unresolved challenge in the field of robotics. The integration of human–robot collaboration, which uses the distinct capabilities of humans and robots while mitigating any perceived threats, might present a viable solution to this problem.

For human–robot interfaces to be effective, they must, at a minimum, represent information that is understandable by both humans and robots over different scales. Hierarchical scene graphs have recently emerged as a powerful and human-understandable way of representing complex 3D environments. These describe environments through a layered or hierarchical graph where the nodes represent different spatial concepts (from low-level geometry to higher-level scene-scale reasoning) and the edges between them represent relationships.

We developed the notion of a scene graph for an agricultural outdoor environment in our prior work Osiris (Mukuddem and Amayo, 2024), which, while powerful, allowed only the construction of a scene graph in an open loop from a single robot. In this work, we specifically investigate the formulation of a scene graph for precision agricultural tasks such as robotic navigation, mapping, and interaction. We demonstrate that the flexibility of our approach allows the hierarchical scene graph to be applied to a variety of precision agricultural needs.

We summarise the main contributions in this study as follows:

- We modify the 3D scene graph structure of *Osiris* to more explicitly encode and allow precision agricultural tasks through the inclusion of a *robot* layer.
- We demonstrate the utility of this flexible formulation across navigation, mapping, and interaction tasks.
- We validate and evaluate the performance of this formulation using real-world data collected in agricultural settings.

The remainder of this article is organised as follows. Related work is discussed in Section 2. An overview of the hierarchical graph formulation is presented in Section 3. Sections 4–6 present the three different variants of hierarchical graphs for precision agriculture, beginning with Osiris-Nav, Osiris-Map, and finally NeurOsiris. Section 7 presents the performance of each of these formulations for different tasks in collected real-world farm data and field experiments. Finally, conclusions are drawn in Section 8.

## 2 Related work

### 2.1 Cooperative robotics in agriculture

There has been a growing interest in robotics research in the agricultural field in the pursuit of optimising efficiency for precision agriculture. Most agricultural robots are task-specific systems designed for tasks such as monitoring, spraying, harvesting, and transportation (Lytridis et al., 2021) with varying levels of autonomy. Cooperative robotics refers to the process in which humans and robots act as a team to achieve a goal and is highly dependent on effective exchange of information between robots and humans (Benos et al., 2023). Particularly in the agriculture sector, collaborative human–robot systems have the potential to enhance productivity (Vásconez and Auat Cheein, 2022) and improve the quality of services (Benos et al., 2023). However, the use of cooperative robotics in the agricultural sector is an emerging trend (Lytridis et al., 2021).

Conventional methods for human–robot interaction in agriculture typically involve devices such as keyboards, mice, pens, and touch screens (Lytridis et al., 2021). These interfaces require detailed inputs, for example, providing GPS coordinates for tasks such as transportation. However, a significant drawback is that the users must possess certain technical skills, which are often lacking in Africa (Lytridis et al., 2021). To address these limitations, vision-based interfaces have been explored for human–robot collaboration (Moysiadis et al., 2024; Tagarakis et al., 2021; Aivazidou and Tsolakis, 2023). Moysiadis et al. (2024) created a system that utilises vision technology to interpret various human gestures and translate them into corresponding robot actions, thereby facilitating coordination between humans and robots in farming settings. This system was successfully tested in an actual orchard setting; however, it faced challenges such as difficulty in consistently detecting the intended human gestures in the agricultural field and the need for meticulous obstacle-avoidance programming as it relied on a complete GPS-enabled robotic operating system navigation stack.

### 2.2 3D scene graphs

3D scene graphs have recently emerged as a powerful and human-understandable way of representing complex 3D environments (Mukuddem and Amayo, 2024). These represent environments through a layered or hierarchical graph, where nodes correspond to different spatial concepts (ranging from low-level geometry to higher-level scene-scale reasoning) and the edges between them represent relationships (Hughes et al., 2022). The choice of layers in the graph and the attributes of its nodes is based on the structure of the environment and is designed with consideration of task- and motion-planning queries (Hughes et al., 2023). Most work on 3D scene graphs has been developed for indoor scenes, but Mukuddem and Amayo (2024) created a 3D scene graph for an outdoor agricultural environment. Chang et al. (2023) developed a 3D scene graph system that takes inputs from multiple robots simultaneously to generate a single coherent 3D scene graph. In this work, we adapt the open-loop 3D scene graph structure developed by Mukuddem and Amayo (2024) to accommodate more specific precision agricultural tasks.

## 3 Osiris overview

This work demonstrates a flexible and adaptable hierarchical graph system that is responsive to different sensor modalities and precision agricultural tasks performed by mobile robots. In particular, we introduce three variants encapsulated within this framework.

-
Osiris−Nav : A hierarchical graph system that enables real-time multi-robot coordination and creation of a hierarchical scene graph from visual input.
-
Osiris−Map : A hierarchical graph system that enables high-precision mapping of agricultural areas using LiDAR and inertial sensors.
-
Neuro−Osiris : A hierarchical graph folded into a neural field for high-resolution interaction of agricultural areas using visual input.

**FIGURE 1:**
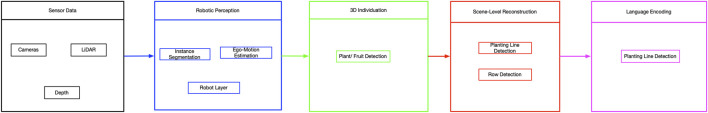
Process flow.

Although the specific construction details of each variant may differ, they share a common construction flow, as shown in Figure 1. The following section details the construction of the common hierarchical graph, breaking it down layer by layer.

The layers of *Osiris* ^*++*^ include farm sections, rows, planting lines, plants, *robots*, and a 3D perception layer. *Layer 1* is a perception layer that captures the environment, and *Layer 2* is a topo-metric graph of the robot and the selected sensor data for precision robotic tasks. *Layer 3* is a subgraph of the plant objects detected within the farm. *Layer 4* is a subgraph of the planting lines. Planting lines represent the lines in which the crops are planted. *Layer 5* is a subgraph of the rows within the farm. A row is defined as a robot-navigable area located between two planting lines; in this way, two planting lines form the lower layer of a specific row. *Layer 6* is a subgraph representing the sections within the farm. Sections are areas of the farm in which crops are of a specific type as a farm can contain multiple types of crops.

Formally defined, the hierarchical graph obtained through *Osiris* ^*++*^ is $O^{++}=\left(\mathrm{V,E}\right)$ , where V is the set of nodes and E is the set of edges between nodes V . The set of nodes V can be divided into the six layers of the graph as follows: $V=\cup_{i=1}^{6}V^{i}$ .

### 3.1 Perception layer

*Layer 1* of the *Osiris* ^*++*^ scene graph is a 3D perception layer and captures the environment at the resolution required for the particular navigation task. This layer is intentionally designed to be flexible and can be represented using a mesh, point cloud, or neural point cloud. This flexibility allows for computing to be traded off when perception accuracy is not very important. An example perception layer is detailed for each precision agricultural task in its corresponding section.

### 3.2 Robot layer

The robot’s movement within the agricultural environment is encapsulated in the robot layer, *Layer 2*. This layer is generated while the robot traverses the agricultural environment and contains a spatio-temporal graph. This layer is characterised as an undirected graph consisting of the pose and spatial vertices $V^{2}=\left\{X,S\right\}$ and their temporal and spatial edges.

Pose vertices correspond to the initial position of the robot, and its ego-motion estimates are sub-sampled regularly to create keyframes. Formally, each pose vertex is defined as $X_{i}^{n}=\left\{R_{i}^{X},t_{i}^{X},I_{i}^{X},D_{i}^{X}\right\}$ , where i is an incremented index as a new keyframe is observed and n is a unique identification number given to each robot to facilitate further localisation tasks. Temporal edges are created between vertices, representing **SE**(3) transformations derived from odometry data, and they closely resemble the classical pose graphs commonly utilised in **SLAM**.

### 3.3 Object individuation layer

An object detection algorithm is used in *Layer 1* to obtain the objects in *Layer 3*. Objects are detected and updated upon the addition of a new keyframe. The specific implementation of this layer varies by task.

### 3.4 Planting line layer

The construction of the first three layers of the scene graph only considers the information introduced in each keyframe. However, as proposed by Mukuddem and Amayo (2024), the detection of planting lines considers the entirety of the objects in the scene. This is the first task in the *Osiris* ^*++*^ pipeline that reasons at the entire scene level, rather than solely at the keyframe level. This is important as the system is required to extract the underlying structure of the row-crop systems. Planting lines are, therefore, generated from the object nodes $V^{3}$ belonging to *Layer 3*.

A two-dimensional (2D) bird’s-eye view of plant objects is obtained as a preprocessing step to better understand the structure of row-system crops. This top–down perspective not only reveals the underlying structure of the crop rows but also simplifies the detection of planting lines. By reducing the problem to a 2D multi-line estimation task, the computational complexity is significantly decreased. A reduced set, $P=\left[p_{1},p_{2},\ldots ,p_{N_{o}}\right]$ , is created, where $N_{o}$ is the number of plant objects, $p_{i}=\left[x_{i},y_{i}\right]$ , and $x_{i}$ and $y_{i}$ are the coordinates of the plant objects found in the original set $V^{3}$ .

Planting line detection is reduced to the fitting of multiple geometric line models to the set of plants P . The convex relaxation algorithm (CORAL) (Amayo et al., 2018) can be used for this. CORAL is a method to fit multiple geometric models to multi-structured data via convex relaxation (Amayo et al., 2018). The result of CORAL is a set of labels L , which assigns each plant object in the set P to a geometric line model $M=\left[M_{1},M_{2},\ldots ,M_{N_{l}}\right]$ , where $M_{i}$ is the 2D equation of a line and $N_{l}$ is the number of labels.

CORAL approaches the multi-model estimation as an energy minimisation problem. In this way, an energy functional that aims for a solution that is geometrically accurate, spatially smooth, and compact is created, as shown below:

$$E\left(L\right)=\overset{N_{l}}{\underset{l=1}{\sum }}\int_{\Omega }\rho_{l}\left(P,\phi_{l}\left(P\right)\right)d\Omega +\lambda \overset{N_{l}}{\underset{l=1}{\sum }}\int_{\Omega }\omega_{N}R\left(\nabla_{N}\phi_{l}\left(P\right)\right)d\Omega +\beta N_{l}.$$ (1)

The initial term of this energy functional represents the geometric cost of assigning a plant object to a particular line model. Here, $\phi_{l}\left(P\right)$ represents the label currently assigned to the object. The residual function $\rho_{l}\left(P,\phi_{l}\left(P\right)\right)$ gives the Euclidean distance between the plant object’s position and the geometric line model $M_{l}$ . It, therefore, penalises label assignments that poorly correspond to the underlying data.

The second term of the energy functional represents a smoothness cost. We expect that in rowed crops, plants that are physically close together have a high probability of belonging to the same planting line. The function R is formulated to penalise neighbouring plant objects that do not share the same label. This is calculated using $\nabla_{N}$ , which calculates the gradient of the current label assignment between neighbouring plant objects. λ trades-off between the smoothness and the geometric cost, while the weighting function $\omega_{N}$ allows for finer control of the influence of neighbouring points; in this case, weighting is reduced as the distance to a plant’s neighbour increases. Here, N is the number of neighbours that every plant object will have.

The final term promotes compactness by favouring label assignments that explain the data using as few planting line models as possible. This reduces redundant models and ensures that the solution more closely resembles the underlying structure. The value β trades off compactness against smoothness and geometric cost.

The optimal label assignment L can be obtained through the minimisation of Equation 1. CORAL (Amayo et al., 2018) can simultaneously minimise the geometric, smoothness, and compactness terms. CORAL is defined Equations 2, 3:

$$\int_{\Omega }|\nabla \phi_{i}\left(u\right){|}_{p}d\Omega =\underset{\Psi_{i}\left(u\right)}{\max}\int_{\Omega }\nabla \phi_{i}\left(u\right)\cdot \Psi_{i}\left(u\right)d\Omega ,$$ (2)

$$s.t.\;|\Psi_{i}\left(u\right){|}_{p^{*}}\leq 1,$$ (3)

where $\Psi_{i}\left(u\right):\Omega \to R^{2}$ is known as the dual function of $\phi_{i}\left(u\right)$ and both $|\cdot {|}_{p}$ and $|\cdot {|}_{p^{*}}$ are dual norms. The optimal values of ϕ(⋅) and Ψ(⋅) are obtained from the first-order primal-dual optimisation.

CORAL presents a solution that is geometrically sound, spatially smooth, and free of redundant labels. Additionally, due to its highly parallel nature, CORAL’s time performance does not reduce significantly even as the number of plant objects within the scene $N_{o}$ increases. This allows it to reason over large scenes without incurring a large time penalty (Mukuddem and Amayo, 2024).

Planting lines are, therefore, detected through CORAL on all plant objects within the scene after every keyframe has been processed. The corresponding labelling L presents edges between the plant object nodes $V^{3}$ and the line models M , which create line model nodes. Line model nodes $\upsilon_{i}^{4}=\left(x_{i},y_{i},z_{i}\right)$ are, thus, added to the set of nodes $V^{4}$ and added to the scene graph O . To obtain $\left(x_{i},y_{i},z_{i}\right)$ , the mean of all the positions of plant objects’ nodes assigned to the corresponding line model is used.

### 3.5 Row layer

Within this framework, rows are defined as traversable sections that provide access to planting lines and, consequently, the planted objects. Rows, therefore, occur between planting lines, and the geometric line models M obtained while extracting the plant line nodes in *Layer 4* can be used for the row detection.

Given a pair of line models, $M_{i}$ and $M_{j}$ , a gradient check is first performed to ensure that they are within a tolerance threshold $t_{\|}$ of being parallel. If the gradient check is within this threshold, the distance between the adjacent planting lines is calculated. A row node, $\upsilon_{i}^{5}=\left(x_{i},y_{i},z_{i}\right)$ , is then added between two planting lines if the calculated distance falls within a specified range, which is determined by the minimum and maximum tolerance values that are informed by the physical structure of the farm. Edges between this node $\upsilon^{5}$ and the two plant line nodes $\left(\upsilon_{i}^{4},\upsilon_{j}^{4}\right)$ are also added to the graph. To obtain $\left(x_{i},y_{i},z_{i}\right)$ , the mean of all the positions of plant line nodes that are assigned to the row node is used.

It is important to note that in this way, a plant line node $\upsilon^{4}$ can belong to more than one row node $\left(\upsilon_{i}^{5},\upsilon_{i}^{5}\right)$ . This differs from most definitions of hierarchical graphs, where current approaches typically ensure that each node at a lower layer has only a single edge to a node in the layer above it (Strader et al., 2024; Hughes et al., 2022; Hughes et al., 2023; Rosinol et al., 2021). Enforcing this would create a level of duplication when dealing with shared spatial concepts, which does not faithfully represent the underlying structure. By retaining the concept of shared spatial concepts in a manner similar to that observed in S-graphs (Bavle et al., 2022; Bavle et al., 2023), where ‘walls’ can be shared by ‘rooms,’ *Osiris* can maintain a high-fidelity representation of the underlying environment.

### 3.6 Section layer

The scene graph consolidator then creates the final layer by consolidating the row nodes $V^{5}$ into a section node $\upsilon_{i}^{6}=\left(x_{i},y_{i},z_{i}\right)$ that is added to the set of nodes $V^{5}$ corresponding to the section layer and the scene graph O . Edges between this node and the row nodes are also added to the graph.

## 4 Osiris-Nav

This section describes the concept and construction of a 3D scene graph that not only enables the integration of multiple sessions and multiple robot inputs but is also primed for robot navigation. Vision-based navigation remains an extremely favourable option for mobile robots and has been widely used for autonomy in various fields. This, coupled with relatively low cost of cameras compared to other sensors, makes them a prime candidate for precision agricultural tasks. Osiris-Nav extensively uses image-based techniques to construct a 3D scene graph that advances towards this goal.

As mentioned in Section 2, each variant of ${\text{Osiris}}^{++}$ only modifies the three base layers, and following object detection, all the subsequent layers of the hierarchical graph remain the same.

### 4.1 Perception layer

Building upon this, the perception layer, namely, *Layer 1*, of Osiris-Nav is developed in real-time from an image data stream, which is subsequently transformed into a 3D metric-semantic mesh. A keyframe-based construction approach was utilised to enhance system performance and ensure the usability of the resulting system in robotic applications. From the image data stream, ORBSLAM3 (Campos et al., 2021) is used to estimate the ego-motion of the camera. Upon detecting a significant alteration in motion, a new keyframe is introduced to incorporate the updated information of the scene. Following the extraction of keyframes, depth-estimation using a depth network and semantic labelling are pursued.

A two-stage approach was employed for instance segmentation. It consists of prompt-guided bounding-box detection, followed by high-quality semantic segmentation within the bounding boxes. Agricultural environments contain a wide range of objects, some of which are directly relevant to agricultural tasks. To effectively handle this diversity, Osiris-Nav leverages a prompt-guided approach. This approach facilitates generalisation, allowing the system to accurately represent rowed crops in a semantic mesh. Furthermore, it ensures that the graph representation is selective, including only objects relevant to agricultural tasks while filtering out irrelevant elements, thereby maintaining a focused and efficient representation of the agricultural environment (Mukuddem and Amayo, 2024).

Grounding DINO (Liu et al., 2023) was used to obtain bounding boxes using keywords such as ‘fruits,’ ‘plants,’ ‘bushes,’ and ‘paths.’ These bounding boxes were fed into “Towards Real-Time Segment Anything” (Wang et al., 2023) for semantic segmentation of stereo images. The resulting semantic images and depth images were combined to create a semantically labelled point cloud. This point cloud is then used with Voxblox (Oleynikova et al., 2017) to generate a truncated signed distance field (TSDF) and Euclidean distance field (ESDF) using data within a certain radius of the robot. The 3D metric semantic mesh is extracted using Voxblox’s (Oleynikova et al., 2017) marching cube implementation.

### 4.2 Robot layer

The robot’s movement within the agricultural environment is encapsulated in the robot layer, namely, *Layer 2*. This layer is generated while the robot traverses the agricultural environment and contains a spatio-temporal graph. In Osiris-Nav, the pose vertices in this layer correspond to the keyframe positions obtained through ORB-SLAM and the associated image and bag-of-words descriptors from the keyframe. The addition of the image and descriptors is critical for enabling the localisation, subsequent aggregation, and autonomy.

Similar to Kimera-Multi (Tian et al., 2022), Osiris-Nav augments the pose graph with a sub-sampling of the semantic mesh in *Layer 1*, allowing joint optimisation of both the pose graph and the underlying mesh. Joint optimisation counteracts both odometry drift due to inaccuracies in visual odometry over long distances and the incorporation of multiple robot scene graphs into a singular coherent scene graph.

The sub-sampled mesh is then simplified using a vertex clustering method that stores these vertices in an octree and then merges vertices belonging to the same voxel as the map grows. These merged vertices thus become the spatial vertices of the robot layer and are immediately connected to the pose vertex of its associated keyframe through a spatial edge. Each of these spatial vertices is defined as $S_{k}=\left(R_{k}^{S},t_{k}^{S}\right)$ , where $R_{k}^{S}\in SO\left(3\right)$ , which is initialised to the identity, and $t_{k}^{S}\in R3$ is the 3D position of the merged mesh vertices.

#### 4.2.1 Robot atlas

The atlas integrates a virtually unlimited number of scene graphs generated from multiple robots and sessions. The atlas then establishes connections between scene graphs that observe the same location. This capability is enabled by a database of DBoW descriptors within the atlas, populated from the robot layers of each scene graph, thus facilitating place recognition across the entire collection of open-loop scene graphs.

This centralised place recognition database, which is continuously updated with new robot layer pose vertices, enables the creation of a single, consolidated, though unoptimised, scene graph through factor graph optimisation using GTSAM (Dellaert, 2012). This is achieved by adding edges between robot layers that observe common regions, which are identified through DBoW descriptor matching, to create the following function for minimisation.

$$\hat{X}=\mathrm{arg min}\overset{n_{s}}{\underset{n=1}{\sum }}\overset{n_{p}-1}{\underset{i=1}{\sum }}\underset{\text{Robot Layers}}{\underbrace{R\left(X_{i}^{n},X_{i+1}^{n}\right)}}+\underset{\alpha_{i},\beta_{j}\in L}{\sum }\underset{\text{Scene Graph Atlas}}{\underbrace{R\left(X_{i}^{n}\alpha X_{j}^{\beta }\right)}}.$$ (4)

Optimisation of the objective function reveals a target set of optimised poses $\hat{X}$ . R is the residual function between two poses, $n_{p}$ is the number of poses in a particular scene graph, and $n_{g}$ is the total number of scene graphs to be aggregated. α and β refer to different scene graphs over which the scene graph atlas detects a common region.

Similar to Kimera-Multi (Tian et al., 2022), once the target poses are obtained, local optimisation is performed to deform the underlying mesh, ensuring accurate alignment with the trajectory defined by the target poses. We direct the reader to Tian et al. (2022) for further technical details on implementation.

### 4.3 3D object detection

An object-detection algorithm is applied to the 3D metric-semantic mesh generated in *Layer 1* to obtain the objects in *Layer 3*. Objects are detected through Euclidean clustering (Rusu and Cousins, 2011) of mesh vertices that were updated on the addition of the new keyframe. The Euclidean clustering algorithm groups together vertices that are within a specified distance threshold, effectively identifying individual objects within the scene. A key variable used in Euclidean clustering (Rusu and Cousins, 2011) is *cluster tolerance*, which relates to the minimum allowed distance between vertices. Vertices are joined together if they are less than *cluster tolerance* apart. The optimal value for the *cluster tolerance* parameter is determined empirically through experiments conducted on the robot within its operational environment.

Euclidean clustering is further restricted to semantic classes corresponding to agricultural produce as defined during the construction of *Layer 1*. This ensures that only plant-related objects are identified and propagated through the scene graph. The clustering process yields a set of vertex clusters, each of which is associated with a bounding box and its centroid. For each object obtained, the centroid position $\upsilon^{3}=\left(x_{i},y_{i},z_{i}\right)$ is used to create the object node and is added to the set of nodes $V^{3}$ representing the object layer. At the same time, an edge is added between the centroid of the boundary box and the clustered vertices of the detected object. Finally, when objects are detected, edges are added between the object centroids and the pose vertices of the robot layer.

Any updates to the pose vertices through optimisation of the scene atlas will trigger a corresponding update to the positions of the object nodes to match the pose vertices. If this optimisation results in overlapping object nodes associated with the same semantic label, the nodes are reconciled into a single node representing their union, ensuring that agricultural entities are not duplicated in the graph.

## 5 Osiris-Map

This section describes the concept and construction of a 3D scene graph that preserves high-precision environmental information while providing users with a human-understandable representation. Osiris-Map leverages a combination of LiDAR and inertial sensors to create its perception and robot layers. The accuracy of LiDAR significantly exceeds that of visual sensors, particularly in outdoor environments, making it a natural choice for high-precision mapping, especially as the agricultural environment expands.

### 5.1 Perception layer

The perception layer, namely, *Layer 1*, of Osiris-Map is developed in real-time from a stream of laser and inertial data, which is subsequently transformed into a 3D semantic point cloud. From the combined streams, Fast-LIO2 (Xu et al., 2022) is used to estimate the ego-motion of the laser. For instance segmentation, an adapted version of SegMatch (Dubé et al., 2017) was used for plant identification. Unlike in Osiris-Nav, where a flexible segmentation target can be used, Osiris-Map relies on naturally delineated objects such as trees in agricultural regions. A similar keyframe approach is used, however, to accumulate consecutive point clouds into a keyframe point cloud.

### 5.2 Robot layer

Similar to Osiris-Nav, the pose vertices in Osiris-Map’s robot layer correspond to the keyframe positions obtained through LiDAR odometry. These pose vertices are enriched with a density image of the corresponding keyframe point cloud with a view to enabling further localisation. The creation of the density image follows the approach proposed in MapClosures (Gupta et al., 2024). While SegMatch segment descriptors are useful for place reconstruction in many robotic scenes, as demonstrated in SegMap (Dubé et al., 2018), their performance deteriorates when applied to the homogeneous scenes common in agricultural environments, which often contain imperceptible differences between plant instances. Density images formed by accumulating multiple point clouds into a single projection are more robust compared to these as they not only capture individual instances but also scene-level relationships.

For Osiris-Map, a bird’s-eye view B projection of the keyframe point cloud was created and then discretised using a voxel grid. This projection B contains points in the bounds shown in Equation 5:

$$\left[\begin{array}{c} x^{\max}\\ y^{\max} \end{array}\right].=B_{\max};\left[\begin{array}{c} x^{\min}\\ y^{\min} \end{array}\right].=B_{\min}.$$ (5)

The discretisation creates a grid $N\left(u,v\right)\in N^{W\times H}$ of resolution $v_{\mathrm{res}}$ metres per cell, with W as shown in Equation 6:

$$W=\left[\frac{x^{\max}-x^{\min}}{v_{\mathrm{res}}}\right];H=\left[\frac{y^{\max}-y^{\min}}{v_{\mathrm{res}}}\right].$$ (6)

With each cell in the grid storing the number of points post-discretisation, this allows for the definition of the greyscale density image I as shown in Equation 7:

$$I\left(u,v\right)=\frac{N\left(u,v\right)-N_{\min}}{N_{\max}-N_{\min}}.$$ (7)

#### 5.2.1 Robot atlas

In Osiris-Nav, the scene graph atlas was powered by a database of DBoW descriptors from keyframe RGB images. In Osiris-Map, this formulation is adapted to use ORB (Oriented FAST and Rotated BRIEF) features for place recognition. These features allow for place recognition across all keyframes presented to the atlas. Following place recognition, the pose-graph constructed in Equation 4 can be optimised to produce a novel set of refined poses.

Unlike Osiris-Nav, which required mesh deformation after optimisation, Osiris-Map can be regenerated on the fly without further optimisation by transforming keyframe point clouds to their updated positions.

### 5.3 3D object detection

A similar Euclidean clustering approach is used for the detection of objects from the semantic point cloud produced in *Layer 1* to obtain the objects in *Layer 3*. Unlike Osiris-Nav, this process operates on the semantic point cloud rather than on mesh vertices, grouping together points that fall within the specified distance threshold.

The clustering process similarly yields a set of point clusters, each associated with a bounding box and its centroid. For each object obtained, the centroid position $\upsilon^{3}=\left(x_{i},y_{i},z_{i}\right)$ is used to create the object node and is added to the set of nodes $V^{3}$ representing the object layer. At the same time, an edge is added between the centroid of the boundary box and the clustered vertices of the detected object. Finally, when objects are detected, edges are added between the object centroids and the pose vertices of the robot layer. After optimisation, object-node reconciliation is carried out similarly to Osiris-Nav, ensuring that trees or plants in the scene graph are not duplicated.

## 6 NeurOsiris

Camera-based approaches, while powerful, often struggle with lighting conditions when creating dense reconstructions of the traversed areas. LiDAR-based approaches, on the other hand, provide more accurate information for the reconstruction of large-scale agricultural environments, as demonstrated in Osiris-Map, but their measurements are both sparser than camera images and devoid of colour information. These limit further interaction with reconstructions as they do not accurately reflect the observed scenes.

There has been a surge of popularity in the use of neural radiance fields (Mildenhall et al., 2021) for reconstructions, with approaches such as SiLVr (Tao and Fallon, 2025) and MegaNerf (Turki et al., 2022) showing the potential for the creation of large-scale, accurate, and interactable reconstructions. In this section, we present a novel formulation, NeurOsiris, which combines the reconstruction capabilities of modern natural radiance fields with a generated scene-graph to enable further interaction.

### 6.1 Perception layer

The perception layer, namely, *Layer 1*, of NeurOsiris is developed offline using a combination of visual inputs and LiDAR information. This differs from the incremental Osiris-Nav and Osiris-Map, in which the entire scene information is available for constructing the perception layer, which in this case consists of a trainable neural network for rendering. The rendering process for each pixel from an image of known pose follows an integral equation that is approximated using quadrature as follows:

$${\hat{c}}_{u}=\overset{N}{\underset{i=0}{\sum }}w_{i}c_{i}.$$

This expression calculates pixel colour as the sum of radiance values along the ray, weighted by the opacity of each point along the ray, with the weights calculated using:

$$w_{i}=exp\left(-\overset{i-1}{\underset{j=1}{\sum }}\delta_{j}\sigma_{j}\right)\left(1-exp\left(-\delta_{i}\sigma_{i}\right)\right).$$

The colours $c_{i}$ and densities $\sigma_{i}$ are obtained by querying the neural network at discrete points along the ray.

In classical nerf models, the network is trained solely using a photometric loss between the rendered and ground-truth images. Photometric loss alone is insufficient for high-fidelity 3D reconstruction; therefore, we utilised a LiDAR depth-based supervision loss to encourage densities along rays to form unimodal distributions that peaked around surface intersections, avoiding the commonly occurring ‘floaters’ when this supervisory signal is absent. We adopted the depth loss used in urban radiance fields (Rematas et al., 2022):

$$L_{\mathrm{depth}}=E_{r∼D}\left[\int_{t_{n}}^{t_{f}}w\left(t\right)-\delta \left(t\right)dt\right].$$

Since the ideal distribution for the density value along a ray is a Dirac delta centred at the depth measurement, the loss measures the deviation of the function from this ideal distribution. To make this computationally tractable, the Dirac delta is approximated using a normal distribution, and the integral is split into three sections to ensure that samples outside the depth value contain no 3D surface.

Our method utilises Nerfacto as a base model, which utilises hash-grid encoding to ensure fast training and generate a neural point cloud. As a single network usually does not have sufficient capacity, we follow the method of Tancik et al. (2022) and partition the scene into larger blocks for which point clouds can be individually extracted and fused to generate the complete perception layer. Similarly to language embedding radiance fields (LERF), we used the ADAM optimiser for the proposal networks and fields with weight decay 10^−9^ and an exponential learning rate scheduler from 10^−2^ to 10^−3^ over the first 5,000 training steps, followed by another 10,000 training steps.

### 6.2 Robot layer

As mentioned in the section above, the training of the radiance field is conditional on images with known poses. As all images are present, the Global Structure-from-Motion approach (GLOMAP) (Pan et al., 2024) was used to obtain camera poses via bundle adjustment. These poses were scaled using LiDAR odometry to ensure that the renderings maintained the scene scale.

### 6.3 3D object detection

Semantic segmentation of images captured by NeurOsiris reveals object locations in the image plane, and with poses provided by GLOMAP and depths obtained from the LiDAR point cloud, a semantic point cloud of the entire scene can be generated. Euclidean clustering of this scene point cloud reveals the detected objects from which the subsequent scene graph layers can be obtained.

### 6.4 Scene graph grounding

The development of the NeurOsiris system has, thus far, not exhibited significant deviations from the Osiris-Nav and Osiris-Map systems apart from the integration of visual and LiDAR data, which enhances the accuracy and resolution of *Layer 1* within the scene graph. Notably, NeurOsiris incorporates the capability to anchor higher layers of the scene graph into neural rendering. Our methodology parallels that of LERF, whereby we enhance the neural radiance fields (NERF) outputs with a language embedding denoted as $F_{\mathrm{lang}}\left(x,s\right)\in R^{d}$ . This embedding function accepts an input position x and a physical scale s , producing a d -dimensional language embedding that is invariant to the viewpoint, thus synthesising information from multiple perspectives.

LERF adopts a multi-patch approach for language supervision and takes the CLIP embeddings of patches centred at pixels that rays originate from. Although this method is useful for open-vocabulary queries such as “Show me a plant,” it struggles with certain precision agricultural queries such as “show me the plants in the first row.” Therefore, we adopt a different embedding approach, using text embeddings of the numerical value that a certain object has to a line, row, or section. We achieve this by creating a hierarchical semantic image, enhanced with object, line, and row IDs. The text embeddings of these numbers are then used to supervise the language embedding, allowing us to deal more naturally with homogenous scenes and promote further interaction. A further benefit of these numerical embeddings is that, unlike open-world queries, where there is a substantial performance gap between African languages and English (Ojo et al., 2025), the use of Arabic numerals and digits is widespread across Africa. While the context surrounding these specific numerals is still necessary, the key embedding remains largely language-agnostic.

## 7 Experimental evaluation

**FIGURE 2:**
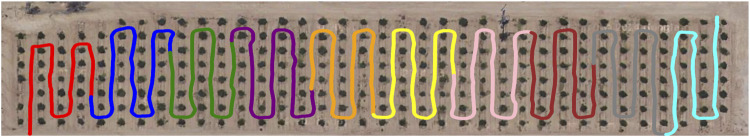
Experimental area of the CitrusFarm dataset. The coloured lines represent the paths along which the robot was driven through the farm.

We design our experiments to evaluate the capability of our system to perform navigation, mapping, and interaction in the context of precision agriculture. To ensure reproducibility and enable fair comparison, we utilise the CitrusFarm dataset (Teng et al., 2023), which is illustrated in Figure 2. This real-world dataset was collected in a commercial citrus orchard using a Clearpath Jackal mobile robot equipped with a Zed2i RGB-D camera and calibrated LiDAR, IMU, and GPS sensors. The dataset is challenging primarily due to its spatial extent: the orchard is approximately 250 m in length, and the traversal distances of the evaluated sequences range from 1.5 to 2 km. Details on the robot and the dataset collection are presented in Teng et al. (2023).

The sequences used for this work’s evaluation were recorded at different times of the day. We consider two separate recordings as two distinct robot input sessions and additionally subdivide one recording into ten partially overlapping segments, as depicted in Figure 2. To evaluate accuracy at higher levels of the scene graph hierarchy, we generate ground-truth annotations by manually segmenting plant instances from the ground-truth LiDAR point cloud data. Axis-aligned bounding boxes were manually placed around individual plant objects by a domain expert using a custom graphical user interface (GUI). The annotated plant instances were subsequently manually grouped into planting lines, which were then aggregated into rows.

### 7.1 Osiris-Nav

The key innovations of Osiris-Nav over the base Osiris introduced in this work were its multi-session performance and its visual navigation capabilities. The following section details their evaluation.

**FIGURE 3:**
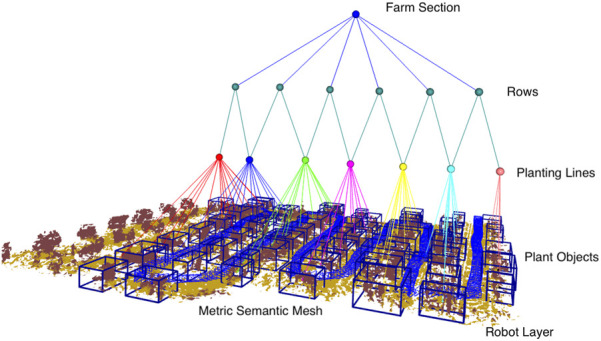
3D scene graph of a citrus farm. The layers of *Osiris-Nav* are (from lowest to highest) are the 3D metric semantic mesh; the robot layer, which enables autonomy; the plant object layer, which identifies plant objects from the mesh; the planting line layer, which groups plant objects into planting lines; the rows layer, which groups two planting lines to form a row, and, finally, the farm section layer, which groups all rows together.

To characterise multi-session performance, particularly to assess the utility of the scene graph atlas, we construct a “ground-truth” single-session scene graph for the full sequences using *Osiris*. We then generate a second scene graph using multiple sessions as inputs to Osiris-Nav; the resulting multi-session scene graph is shown in Figure 3. The multi-session inputs correspond to a subdivision of the full sequence used as input to *Osiris*, as outlined in Figure 2. We compute the metrics described below for both the single-session and multi-session configurations, and we compare these results to quantify the effectiveness of the proposed multi-session capability.

For plant objects in Osiris-Nav, accuracy was calculated by the percentage of objects in the ground-truth annotated point cloud that have an object estimated by Osiris and Osiris-Nav with the correct semantic label within a specified radius (“% found”) and the percentage of objects in the estimated scene graph that have a ground-truth object with the correct semantic label within a specified radius (“% correct”).

**FIGURE 4:**
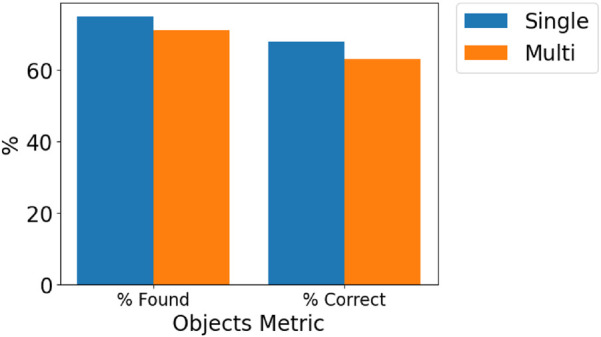
Graph showing objects found and correctly identified objects for a single robot (Osiris) vs. a multi-robot (Osiris-Nav) system.

From Figure 4, it can be observed that *Osiris* achieves adequate levels of objects found and objects correctly identified; however, these results are not exceptional, which can be attributed to the accuracy of depth estimation from the visual sensors. *Osiris-Nav* has a slightly lower number of objects that are both correctly identified and found. This is typical of multi-merging techniques, with a slight penalty incurred, particularly due to overlapping mesh faces at the edges of merged maps, which can result in missed detections.

**FIGURE 5:**
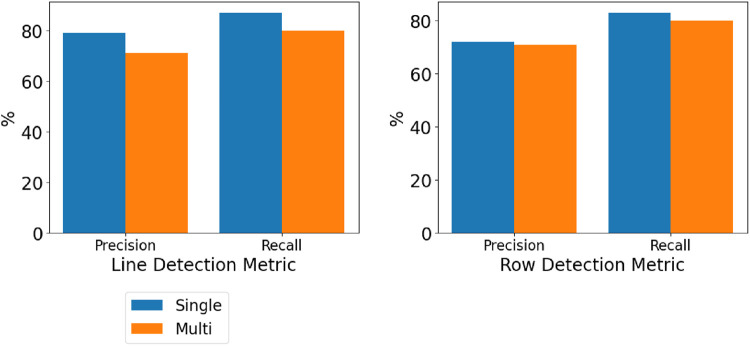
Graph showing line detection precision and recall and row detection precision and recall for a single robot (Osiris) vs. a multi-robot (Osiris++) system. Variable scene-level perception parameters were kept the same for both systems.

However, when higher levels of the scene graph were queried, specifically, lines and rows, as shown in Figure 5, similar values for Osiris-Nav and the base Osiris were reported, indicating that Osiris-Nav accurately merges multiple sessions. Qualitatively, the scene-level perception accurately captures the structure of the environment’, as shown in Figure 3. The variances in the performance of 3D perception layer metrics, compared with those of the scene-level perception layer metrics, indicate that scene graph merging has less impact at higher levels.

**FIGURE 6:**
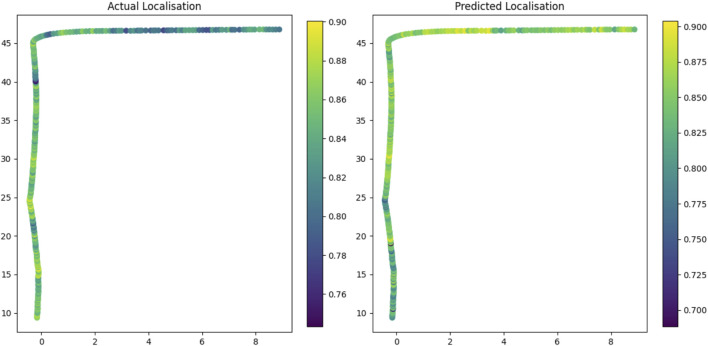
Predicted vs. actual localisation performance using the robot layer.

To assess the autonomy capability, we construct a test pass using sequence 1 from the CitrusFarm dataset (Teng et al., 2023). While the accuracy metrics in Figure 4 suggest that localisation occurs across shared regions, scene reconstruction tasks generally require only sparse localisations. In contrast, navigation—particularly under a visual teach and repeat (VT&R) framework—demands more frequent localisations to minimise drift relative to the teach pass. Adopting the method proposed by Dequaire et al. (2016), we define a localisation envelope to explicitly estimate the localisation performance of the robot layer for VT&R. In this approach, a Gaussian process (GP) is trained to predict the localisation envelope, including in areas that have not yet been revisited. The GP is trained on a sequence from the same citrus farm that overlaps with part of the teach pass, allowing us to test whether the system can reliably localise and provide high-level guidance on the robot’s required orientation to follow the teach path. The results in Figure 6 demonstrate that the scene graph, via its robot layer, can successfully localise during autonomous operation, indicating that the proposed framework can enable ground-robot autonomy in agricultural settings.

### 7.2 Osiris-Map

The key innovation of Osiris-Map over the base Osiris was its retention of higher-precision information for mapping purposes. For the evaluation of Osiris-Map, three sequences from the Field-One dataset, shown in Figure 2, were used. The first sequence consisted of data taken in a lawnmower configuration where every row was traversed once. This was then compared to the second sequence, in which the robot traversed every fifth row, and the third sequence, in which the robot went through every other row and then looped back until all the rows had been traversed.

**FIGURE 7:**
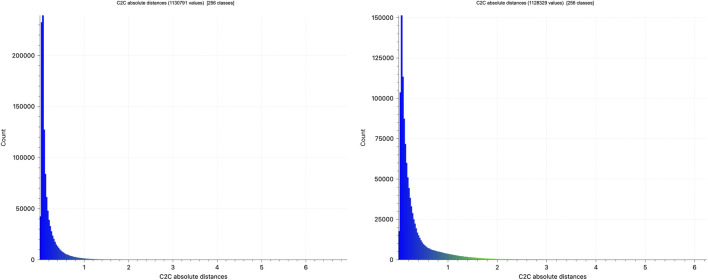
Error histogram between the accumulated point clouds of sequences 2 and 1 on the left, along with sequences 3 and 1 on the right.

The evaluation commenced with an analysis of *Layer 1* of Osiris-Map, specifically, the accumulated point cloud generated throughout the sequence, to assess its precision and its preservation of fine-grained geometric information. The accumulated point clouds from sequences 2 and 3 were thus compared with the point cloud generated in sequence 1 to compute the accuracy metrics shown in Figure 7. The error histograms show that, even across the large observed area, the point clouds generated are largely self-similar, with significant overlap, despite the three divergent sequences starting from different locations.

**FIGURE 8:**
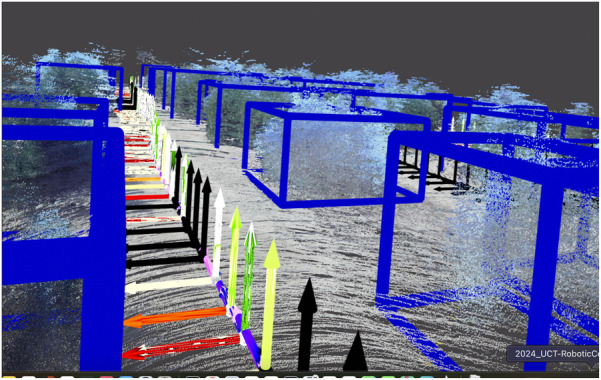
Object detections in Osiris-Map detections. The point cloud generated from Osiris-Map is overlaid with RGB colour information for visualisation. Osiris-Map is able to create a distinct bounding box for each tree in this point cloud to form its *Layer 3*.

**FIGURE 9:**
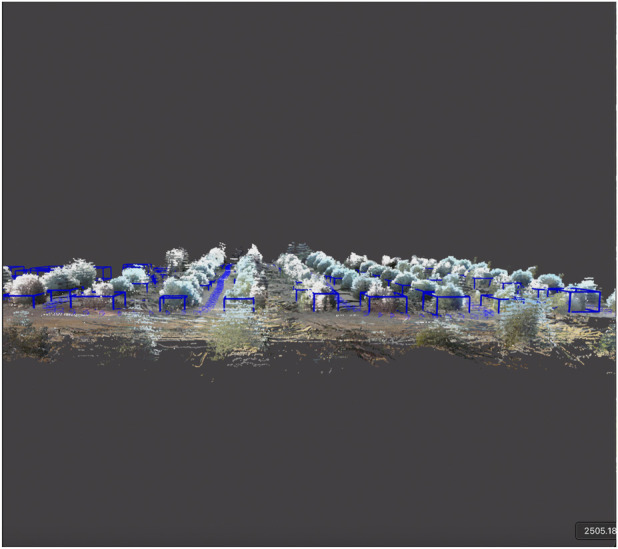
Zoomed-out view of the scene captured through Osiris-Map. In this, the point cloud generated from Osiris-Map is overlaid with RGB colour information for visualisation, with object detection bounding boxes also being showcased.

Attention was then directed to the object detection performance of Osiris-Map, as shown in Figures 8, 9, with the manually labelled accumulated point cloud for each sequence serving as the ground-truth. In all three sequences, only a single tree of the 282 trees in the citrus farm was not detected. While this could be expected in sequences consisting of well-separated citrus trees due to the depth accuracy of LiDAR scans, the low-odometry drift and strong object-merging ability of Osiris-Map were still able to prevent over-segmentation of the scene when approached from different viewpoints and over long loops. The accuracy of the lower levels of Osiris-Map permeated to the higher levels, with perfect precision and recall recorded for the lines and rows layers.

### 7.3 NeurOsiris

The key innovation of NeurOsiris, compared to the base Osiris, lies in its increased photorealism and scene-level grounding. For evaluation of this approach, five samples of rows from sequence 1 of the CitrusFarm dataset were selected, trained, and evaluated.

**FIGURE 10:**
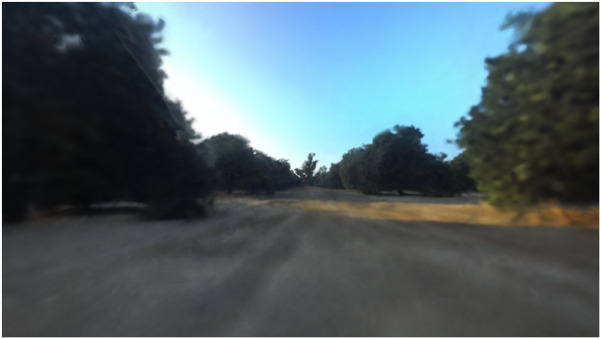
Novel view rendering from NeurOsiris.

To evaluate the photorealism of NeurOsiris, the peak signal-to-noise ratio (PSNR) and structural similarity image measure (SSIM) were used. For NeurOsiris, this resulted in PSNR and SSIM scores of 17.18 and 4.04, respectively, when averaged across the row samples. The base Nerfacto model returned scores of 19.09 and 5.05 across the same scenes. To enable natural-language queries, similarly to LERF, NeurOsiris employs a larger hash grid than pure RGB approaches, which affects its raw photo-realism performance, as shown by the comparison with Nerfacto. However, as shown in Figure 10, the outputs exhibit a significant increase in photo-realism compared to the previous base Osiris variants.

**FIGURE 11:**
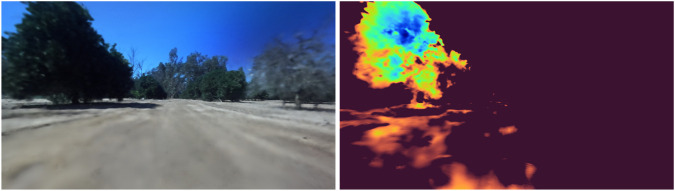
Novel view rendering and the accompanying relevance map for the prompt “Show me the fourth plant.”

**FIGURE 12:**
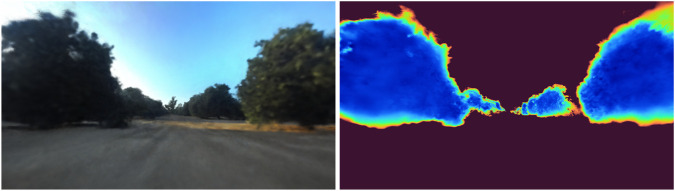
Novel view rendering and the accompanying relevance map for the prompt “Show me the plants in row 1.”

To evaluate language grounding, relevant images of objects and row queries, as shown in Figures 11, 12, were generated for the sample rows. These were compared to the semantic segmentation images used to train NeurOsiris, and the mean intersection over union was calculated. For objects, an mIoU of 67% was achieved, while a slightly higher of 72% was obtained for rows, demonstrating the grounding of the scene graph within the neural rendering.

## 8 Conclusions and future work

In this article, we present a systematic approach that expands hierarchical graph approaches for different precision agricultural tasks. We develop the notion of a flexible scene-graph approach and modify it for navigation, mapping, and interaction tasks while overcoming the challenge of the homogenous nature of agricultural environments. While Osiris++ demonstrates the utility of hierarchical scene graphs for precision agriculture, several avenues still remain for extending its capabilities, particularly in the context of diverse agricultural environments.

### 8.1 Multilingual human–robot interaction

A primary motivation of this work is to lower the barrier to the adoption of agricultural robotics in Africa. Although NeurOsiris uses numerical embeddings to bypass some of the challenges in multilingual queries, it currently can only understand the context around the numerical embeddings in English. Future work will support the expansion of this framework to explicitly accommodate local African languages such as isiXhosa, isiZulu, and Swahili. This will enable field workers to issue commands (e.g., “Ndibonise izityalo kumqolo 1”/”Show me the plants in row 1”) in their native tongue, which is a critical step towards inclusive technology adoption.

### 8.2 Closed-loop autonomy and navigation

In this work, we validated the autonomy capability of Osiris-Nav through predicted localisation envelopes. Future research will transition this to a fully closed-loop navigation system, utilising the VT&R framework directly on the robot hardware. This will involve quantifying real-time path-tracking error and obstacle avoidance performance in dynamic field conditions, moving beyond the current open-loop evaluations.

### 8.3 Spatio-temporal (4D) monitoring

Agricultural environments are highly dynamic, with crops undergoing significant changes over a season. The current system treats the environment as static within a session. We aim to extend the scene graph formulation to a 4D representation that can track individual plant growth, fruit yield, and disease progression over weeks or months. This “long-term memory” would allow the scene graph atlas to not only merge sessions but also highlight temporal anomalies for farmers.

## Data Availability

The original contributions presented in the study are included in the article/Supplementary Material; further enquiries can be directed to the corresponding author.
